# Highly Stable Core-Shell Nanocolloids: Synergy between Nano-Silver and Natural Polymers to Prevent Biofilm Formation

**DOI:** 10.3390/antibiotics11101396

**Published:** 2022-10-12

**Authors:** Ekaterina A. Kukushkina, Helena Mateos, Nazan Altun, Maria Chiara Sportelli, Pelayo Gonzalez, Rosaria Anna Picca, Nicola Cioffi

**Affiliations:** 1 Chemistry Department, University of Bari “Aldo Moro”, Via Orabona 4, 70126 Bari, Italy; 2 CSGI (Center for Colloid and Surface Science), Via Orabona 4, 70126 Bari, Italy; 3 ASINCAR (Research Association of Meat Industries of Principado de Asturias), 33180 Noreña, Spain

**Keywords:** nanocomposite, antimicrobial, silver nanoparticles, chitosan, polymer

## Abstract

Active investment in research time in the development and study of novel unconventional antimicrobials is trending for several reasons. First, it is one of the ways which might help to fight antimicrobial resistance and bacterial contamination due to uncontrolled biofilm growth. Second, minimizing harmful environmental outcomes due to the overuse of toxic chemicals is one of the highest priorities nowadays. We propose the application of two common natural compounds, chitosan and tannic acid, for the creation of a highly crosslinked polymer blend with not only intrinsic antimicrobial properties but also reducing and stabilizing powers. Thus, the fast and green synthesis of fine spherically shaped Ag nanoparticles and further study of the composition and properties of the colloids took place. A positively charged core-shell nanocomposition, with an average size in terms of the metal core of 17 ± 4 nm, was developed. Nanoantimicrobials were characterized by several spectroscopic (UV-vis and FTIR) and microscopic (transmission and scanning electron microscopies) techniques. The use of AgNPs as a core and an organic polymer blend as a shell potentially enable a synergistic long-lasting antipathogen effect. The antibiofilm potential was studied against the food-borne pathogens *Salmonella enterica* and *Listeria monocytogenes*. The antibiofilm protocol efficiency was evaluated by performing crystal violet assay and optical density measurements, direct visualization by confocal laser scanning microscopy and morphological studies by SEM. It was found that the complex nanocomposite has the ability to prevent the growth of biofilm. Further investigation for the potential application of this stable composition in food packaging will be carried out.

## 1. Introduction

The increasing awareness of the problem of antimicrobial resistance and biofilms impacts on everyday life for industrial sites, medical facilities and households and highlights the importance of and need for change from humanity [1]. Annual reports prove the rise in the number of deaths and the increase in the economic burden on countries worldwide due to the problem of antimicrobial resistance and bacterial contamination [2]. Antimicrobial resistance (AMR) is tightly connected and associated with the development and spread of biofilms [3]. When adherent bacteria is tightly attached on the surface and a protective environment called an extracellular polymeric substance (EPS) is secreted around this bacterial aggregate, antimicrobials are much less active or not active at all against pathogens [4]. In the recent years, the search for alternative antimicrobial solutions to fight AMR and biofilm development has been trending due to the high demand from various industrial and social sectors [1,3,5]. The decreasing effectiveness of conventional antimicrobial agents, the time consuming R&D processes for the development of new synthetic formulations and the widespread misuse and overuse of antibiotics are all continuously pushing the research community to work harder on finding new non-conventional, safe and effective antimicrobial agents [1,2,6]. Nowadays, the use of natural compounds is a very promising and popular approach in the design of new bioactive materials. To give an example, biopolymers and polysaccharides extracted from natural sources are used in tissue engineering as drug delivery systems and eco-friendly packaging materials [7,8,9,10]. Chitosan (CS), polysaccharide extracted from crab shells and other widespread sources, is widely used due to its technologically relevant properties and possible applications in various fields [11]. The industrial use of chitosan is attractive due to its cheap price and tunable properties. Its wide range of bioactivity and its ability to work as an anti-inflammatory and antimicrobial additive, make chitosan and its derivatives a potential material for wound healing, dentistry and other biomedical applications [12,13,14,15,16]. Among other natural compounds, chitosan is famous for its film-forming properties and high chemical reactivity towards many other chemicals [17]. However, its relative mechanical integrity under specific conditions and water absorption ability limit its application in certain fields [17]. Several successful attempts to improve its mechanical stability through functionalization and doping have been made. For example, Nataraj and colleagues reported improved water absorption and tensile strength parameters for CS-based biodegradable films upon the addition of citric acid and alkali treatment [18]. Pavoni et al. provided another example of the successful improvement of the physicochemical and mechanical properties of CS films by a common crosslinking agent, glutaraldehyde [19]. This research group also studied the influence of the addition of the crosslinker on biodegradability: the addition of glutaraldehyde (GA) extends the degrading time but does not prevent the process itself. Due to the toxic nature of the crosslinker, the release of GA was evaluated, and it was found that its release was not observed after the incubation period.

Tannins, more precisely tannic acid (TA), is another widespread natural compound which has several attractive features similar to chitosan, with an interesting one in particular being that it exhibits an inhibitory effect against many pathogenic microorganisms [20]. An interesting feature which is beneficial from the point of view of material science is the ability of tannic acid to crosslink chitosan and exhibit reducing and stabilizing properties towards metals such as Au, Ag, Pd and Cu [21]. Several authors have reported the combination of chitosan and tannic acid in order to improve the mechanical properties of chitosan-based materials and also to potentially provide a stronger antimicrobial effect [22,23,24,25]. In fact, Rubentheren et al. reported the improved tensile strength of CS-based film when TA was introduced as a crosslinker [26]. Nevertheless, the performance of CS/TA combinations in humid environments remains limited and has yet to be improved for real-life applications, for example, in the smart film packaging sector as antimicrobial biodegradable packaging material [20]. Due to the availability of several functional groups (amino, carbonyl and secondary and primary hydroxyl groups) which can potentially be involved in reduction processes, chitosan and tannic acid, both individually and in combination, were used for the synthesis of metal nanoparticles [27]. Several metals were successfully embedded in such matrices, including silver; however, for their synthesis, as usual for chemical routes, the introduction of additional chemicals is needed, and the stability of the nanophases over time can be an issue [28].

Metal nanoparticles, and silver in particular, have been well-known for centuries for their antimicrobial and other beneficial properties [29,30]. In the nano state, silver and silver-based materials are able to provide long-term effects and act as a non-conventional antimicrobial agent [31]. With variations in size and shape, bare AgNPs exhibit bactericidal and bacteriostatic action against a wide range of pathogens, including resistant bacteria, viruses and biofilms. One of the challenges related to the use of AgNPs as antimicrobials is related to their potential toxicity and their possible environmental release. The other important point is related to AMR development against metal nanoparticles [32]. Bacteria, constantly adapting to survive in the changing environment, develop several defense mechanisms against NPs. For example, they are able to secrete specific substances which trigger the aggregation of the nanoparticles, thus lowering or even disactivating their antimicrobial effect [33]. Nevertheless, these obstacles can be potentially eliminated if bare AgNPs are combined with other compounds. In a review paper, we recently discussed the potential of combining Ag nanoparticles with natural antimicrobials to prevent resistance development and to improve the properties of individual components [27]. Combining Ag nanoparticles with peptides, polysaccharides and polyphenols has resulted increased antipathogen action, higher stability and prolonged effects. Most importantly, due to several potential inhibitory mechanisms involved, pathogens are less likely to develop resistance when an Ag-based composite antimicrobial is used. Taking into account the intrinsic antimicrobial property of the organic part of the composite, the cationic effect upon direct interaction, the release of Ag ions and the generation of ROS, the combination of all components together in the form of a nanocomposite could potentially have strong bacteriostatic and bactericidal action against a wide range of pathogens, including resistant bacterial strains and biofilms [34,35]. The strong antimicrobial power of nanosilver and the bacteriostatic action of compounds such as chitosan and tannic acid provide the synergy required to prevent resistance development. Notably, Ag-based compositions have proven to be effective against biofilm development, exhibiting an inhibitory effect on mature monomicrobial biofilms [36,37,38]. The combination of nanosilver with organic antimicrobials result in not only antibacterial and antibiofilm action but also antiviral potential. Orlowski and his group reported the development of tannic acid-modified AgNPs with antiviral activities against Herpes Simplex Virus Type 2 [39,40]. The simultaneous use of crosslinked chitosan and tannic acid to reduce and/or stabilize metals and metal oxides, including silver, was reported by several groups, and the final systems were used for various applications, including a catalytic system for electrochemical detection and in synthetic processes [41,42] and as a promising wound dressing material [43].

Based on the abovementioned facts, we propose the combination of a highly crosslinked polymer blend with ultrafine spherical AgNPs, which are arranged during chemical reduction in a core-shell manner. A polymer shell, composed of crosslinked chitosan and tannic acid, acts like a reducing and stabilizing formulation for the synthesis of nanosilver. By performing a fast and easily scalable chemical synthesis, the organic shell tightly protects the AgNPs’ cores, in this way providing long-term colloidal stability and potential prolonged silver ion release with antipathogen potential. The final polymer blend composition was fabricated taking into account the reducing and stabilizing potentials of individual components, and the effect of their presence has been studied. Moreover, by combining two antimicrobial agents with different mechanisms of action against a wide range of pathogenic microorganisms, we aimed to achieve strong and prolonged bacteriostatic, bactericidal and antibiofilm action, possibly avoiding resistance development. In this way, we addressed one of the most challenging tasks related to the development of novel nanoantimicrobials (NAMs), with a potential for application on an industrial scale.

## 2. Results and Discussion

### 2.1. Study of the Formation of AgNPs

**Scheme 1:**
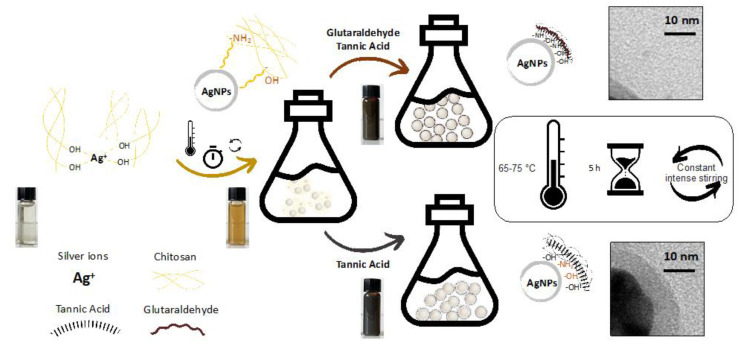
Synthetic scheme of the synthesis of nanocomposites.

The synthetic route in Scheme 1 demonstrates how Ag ions were combined with reducing and stabilizing agents which were used for the development of core-shell nanocolloids as well as the outcome of the combination of them under specific reaction conditions. Sample labelling is introduced in Table 1. For the one-pot synthesis of core-shell nanocomposites, several steps were followed. Three colloidal forms of silver were prepared: CS/AgNPs (1-Ag), CS/TA/AgNPs (2-Ag) and CS/GA/TA/AgNPs (3-Ag). A total of 0.1 M aqueous AgNO_3_ was used as a silver ion precursor. Under constant heating (60 ± 5 °C) and moderate stirring, a 1.5% *w/v* chitosan solution in 1% diluted acetic acid was used as a stabilizing and reducing agent at the initial nucleation stage. In a typical synthesis, 1 mL of silver precursor was introduced to 5 mL of hot chitosan solution in a glass vial, stirred vigorously and left under stirring for 4 h with protection from light. At the end of this initial synthetic step, a color change from pale yellow to bright and intense yellow was observed, indicating the formation of a 1-Ag composite. In the next step, there are two possible ways to proceed. First, only tannic acid was introduced (0.008 mg) and left for 15 min, which formed an 2-Ag composition with a thick organic shell around metal core. Second way involves introducing 0.15 mL of 5% *w/w* aqueous glutaraldehyde solution under vigorous stirring and leaving it for 15 min, then introducing 0.008 mg of tannic acid and keeping it for 15 min. In the first case, a further change in color is observed to grey-brown, and the 3-Ag final colloidal solution has red-brown intense color. The next final step is the introduction of EtOH absolute for the neutralization of the chitosan solution (by bringing the pH closer to neutral values), to promote the solidification of the polymer and the crosslinking of the shell around the metal core. For the additional characterizations and control analysis, a polymer blend solution without AgNPs was developed in the same way, but Milli-Q water was used instead of silver precursor. The flasks were kept under dark conditions throughout the synthesis to avoid any photochemical reactions.

When it comes to the detection of AgNPs after chemical synthesis, it is important to have a rapid and versatile tool to prove their formation and determine their properties. It is well known that antimicrobial activity is directly and highly related to physico-chemical properties such as size, shape and stability. Thus, in the following section, the study of the properties of AgNPs that were freshly prepared by chemical synthesis, then aged and stabilized by different agents (CS, TA and GA), is discussed.

#### 2.1.1. UV-Vis Spectroscopy

The UV-visible spectroscopy was used to characterize the reaction solutions after the synthesis of the AgNPs in the presence of different components and at various concentrations in terms of the precursor. In most studies, a color change from pale yellow to yellow/brown is used as a preliminary indication of AgNP formation in the solution. In the case of this work, the color of the starting solution, the CS solution, was already yellowish, but this did not affect the visual perception of the reaction outcome.

**Figure 1:**
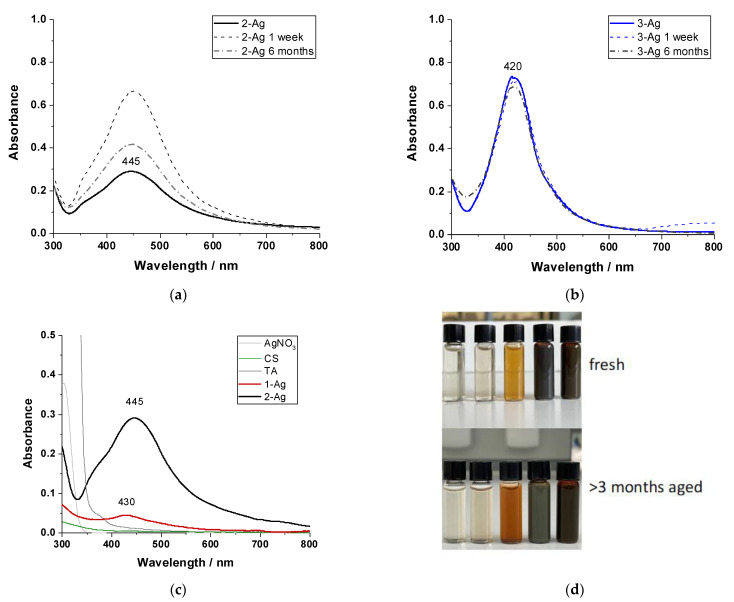
UV-visible spectra of fresh (solid) and aged (dashed, 1 week and 6 months) solutions of colloidal silver synthesized and stabilized by (**a**) CS and TA (2-Ag) and (**b**) by CS, glutaraldehyde (GA) and TA (3-Ag); (**c**) control solutions: silver precursor (AgNO_3_), chitosan (CS), tannic acid (TA) and silver synthesized and stabilized only by CS (1-Ag), by CS and TA (2-Ag). Photos on the panel (**d**) correspond to fresh (upper) and 6-months-aged (bottom) visual appearances of control and colloidal solutions (from left to right): CS (1), CS/GA/TA (3), CS/AgNPs (1-Ag), CS/TA/AgNPs (2-Ag), CS/GA/TA/AgNPs (3-Ag).

**Figure 2:**
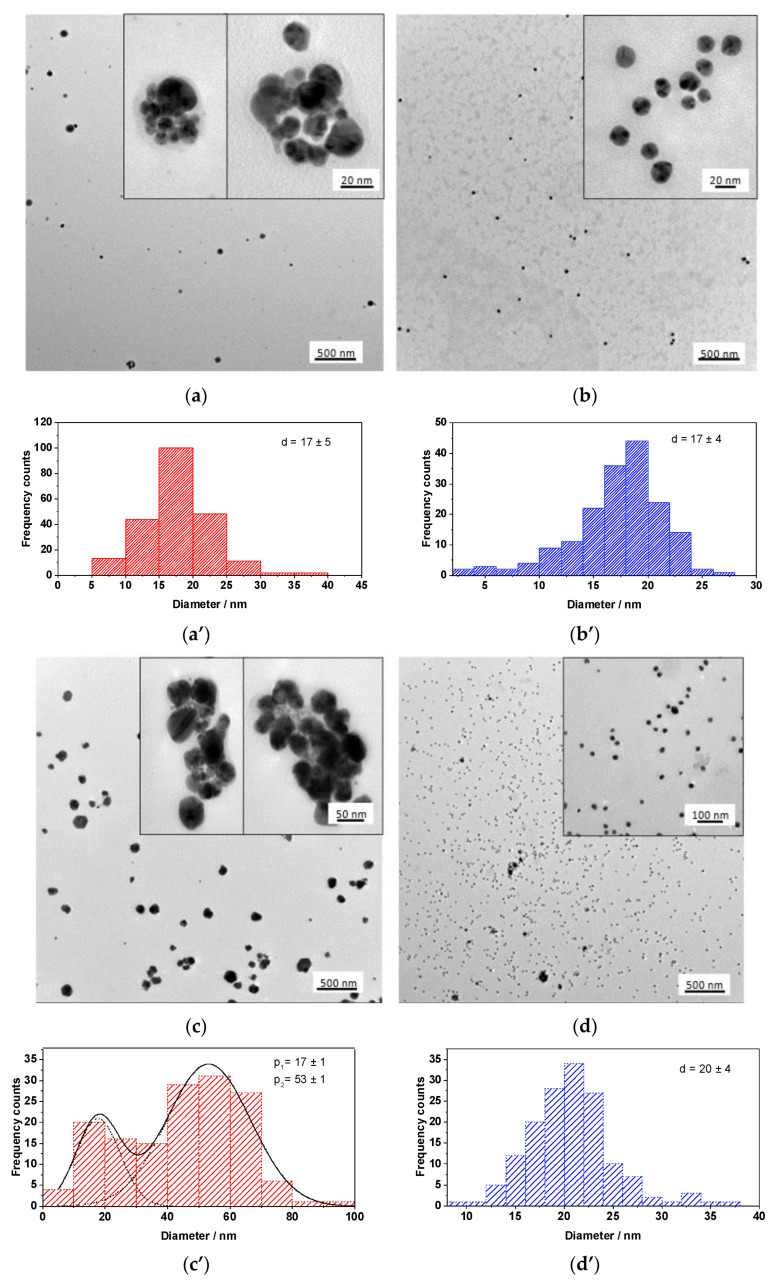
TEM micrographs (**a**–**d**) and corresponding size distribution histograms (**a’**–**d’**) of fresh (**a**) CS/TA/AgNPs and (**b**) CS/GA/TA/AgNPs nanocolloids and 6-months-aged (**c**) CS/TA/AgNPs and (**d**) CS/GA/TA/AgNPs compositions and size distributions.

**Figure 3:**
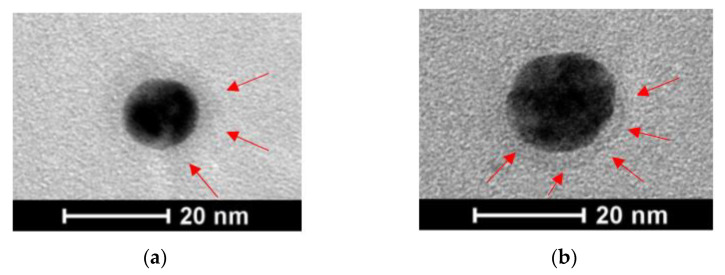
Appearance of thin organic shell around metal nanocore corresponds to (**a**) CS/TA/AgNPs (2-Ag) and (**b**) to CS/GA/TA/AgNPs (3-Ag) individual nanoparticles.

Figure 1a–c shows the UV-vis absorption spectra of the CS (1), CS/TA (2), CS/TA/AgNPs (2-Ag), CS/GA/TA/AgNPs (3-Ag) and reference solutions with no absorbance at the area of interest, AgNO_3_ and TA. The color change throughout the reaction was drastic and observable by the naked eye for all the solutions with different compositions (Figure 1d): the CS neat solution had a pale yellow color, 1-Ag had an intense yellow/orange color and the TA had a dark-grey color, while 3-Ag had an intense dark brown color. The 1-Ag solution exhibited the typical SPR broad band of polydispersed AgNPs at 430 ± 2 nm, while the sample containing TA exhibited a maximum absorbance peak at 445 nm. The SPR absorbance intensity was three times higher for the sample containing 0.05 and 0.3 TA at the same dilution rates of 1-Ag and 2-Ag, respectively. This could be explained by the fact that TA brings additional reducing and stabilizing effects, thus the AgNPs were converted more efficiently from the precursor and appeared to be larger in size. It is worth highlighting that according to the width of the SPR peak, the TA did not drastically increase the polydispersity of the metal nanophases. The intensity rose for samples containing TA at wavelengths below 320 nm, corresponding to the presence of complex phenolic constituents. When GA was introduced, for the resulting 3-Ag sample, the plasmon absorbance rose from 0.3 to almost 0.8 and the shape of the peak also changed, becoming sharper with a blue-shifted maximum at 420 nm. One of the proposed explanations for this is the following: upon the introduction of the additional crosslinking agent, the CS/GA/TA/AgNPs polymer matrix has higher reductive and stabilizing properties and promotes the generation of metal nanophases by the conversion of AgNO_3_ in a more efficient way. This explanation is also supported by the TEM analysis (Figure 2a,b) of individual particles and differences in terms of organic shell thickness (Figure 3a,b) between 2-Ag and 3-Ag. The progressive addition of the three agents with different reduction potentials increased the production of AgNPs, and when all of them were present in the system, in a specific order of addition, the metal precursor was successfully and almost fully converted into AgNPs, resulting in higher absorbance values—1-Ag 0.05, 2-Ag 0.3 and 3-Ag 0.8—at the same dilution rates and a sharper SPR peak, with a shifted maximum from 430 nm to 445 nm and 420 nm for the CS, CS/TA/AgNPs and CS/GA/TA/AgNPs, respectively. The stability experiments (Figure 1a,b, dashed) confirmed this hypothesis: the sample which did not contain the additional crosslinker, 2-Ag, exhibited a time-dependent rise in SPR intensity, which was attributed to the further ongoing reduction process; on the contrary, the 1-week old sample containing GA (3-Ag) did not exhibit a rise in SPR intensity during storage time (ambient temperature, dark place). Colloidal stability and a lack of aggregation were absent for the fresh, 1 week- and 6 months-aged CS/GA/TA/AgNPs (Figure 1b), but in case of the fresh CS/TA/AgNPs (Figure 1a), it is reasonable to hypothesize that, for the selected concentration of the silver precursor (0.1 M) and total reaction time (4 h 30 min), the reductive potential of only CS and TA was not strong enough, and the reduction kept going for days (Figure 1a, dashed) before it reached its highest conversion of Ag^+^ to Ag^0^. By referencing the changes in the colors of the solutions upon storage, as shown in Figure 1d, it is possible to support the results found by UV-vis.

#### 2.1.2. Transmission Electron Microscopy

Transmission electron microscopy (TEM) provided information on the size distribution of the metal nanoparticles and on the role of the organic components. These results demonstrated the importance of the crosslinking degree on the size distribution and stability, e.g., the addition of GA. Moreover, TEM characterization shed light on the shell formation depending on the composition of the polymer blend.

In Figure 2a–d, TEM micrographs of diluted colloidal solutions of CS/TA/AgNPs (2-Ag) and CS/GA/TA/AgNPs (3-Ag) are presented. If we compare the nanoparticles in Figure 2a,b at a higher magnification, we observe agglomerates and polydisperse particles in the first case and monodispersed individual particles in the second. The average size of the spherical particles for both 2-Ag and 3-Ag compositions was found to be approximately 17 nm. Thestability was investigated extended time period (6 months), and a less complex composite exhibited (Figure 2c,c’) the presence of two populations of NPs, with average sizes of 17 and 53 nm. On the contrary, the presence of GA lead to a prolonged colloidal stability and prevented the aggregation of the smaller NPs to bigger clusters.

Taking a closer look at the micrographs, it was possible to observe a thin shell around the metal core for both the CS/TA/AgNPs and CS/GA/TA/AgNPs composites. In the first case, an organic shell was clearly visible around individual particles (Figure 3a) and around agglomerates (Figure 2c), with each having the same thickness of around 6 nm. For the 3-Ag, the organic shell was less visible on TEM micrographs and harder to detect on individual nanoparticles (Figure 3b). It is worth noting that in the areas with a more concentrated population of AgNPs (Figure 2b, at a higher magnification), the mean inter-particle distance was found to be almost the same among all the particles. This can be attributed to a presence of the organic shell, which keeps the particles separated. By measuring the distance between the particles, taking the average and dividing by two, we found the average thickness of the particle shell to be 2.7 nm (Table 2). The thickness of the shell of the individual particle (Figure 3b) appeared to be 2.9 nm, which is in accordance with the measurements of inter-particle distances. This way, it was possible to establish the differences in terms of the organic shell thickness, which is related to the composition. When GA is present, the crosslinking degree and density are higher and the thickness is lower, resulting in tighter shell, which better protects the metal core. It is important to highlight that, according to the literature, the SPR peak of nanoparticles of 20 nm has a max absorbance at 400 nm [44]. In the case of the 2-Ag, we observed an SPR maximum at ~445 nm and the NPs diameter was found to be 18 ± 5 nm, whereas for the 3-Ag, we saw a relative blue shift with a resulting peak at 420 nm for the same size (17 ± 4 nm) of the core-shell nanocomposite. This is another direct indication of the influence and structural and chemical differences of organic shells, which are responsible for a relative red shift in the silver SPR peak, in comparison to bare silver nanophases, which are reported in the literature [44,45].

### 2.2. Investigation of Crosslinking Events and Other Properties of Core-Shell Nanocompositions

#### 2.2.1. Fourier Transform Infrared Spectroscopy

Using the TEM and UV-vis techniques, we determined the influence of the chemical composition, in particular the crosslinking of the polymer blend, on the shell thickness and related properties. In order to investigate how the addition of each individual component influences the chemical structure of the polymer blend, FTIR was used.

**Figure 4:**
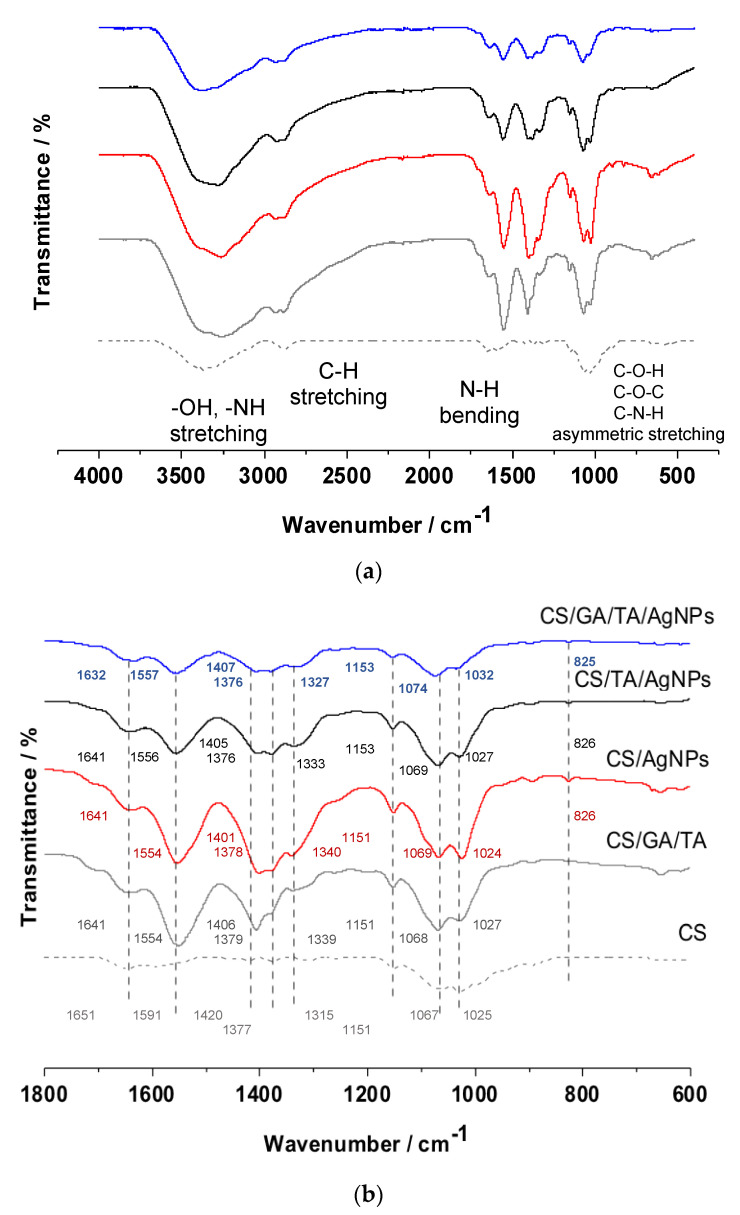
FTIR analysis of individual components and composites of different compositions: (**a**) full spectra with the highlighted regions of interest, (**b**) detailed spectra in the fingerprint region with highlighted changes in the positions of the peak and relative intensities. Dashed grey corresponds to CS (1), grey to CS/GA/TA (3), red to CS/AgNPs (1-Ag), black to CS/TA/AgNPs (2-Ag) and blue to CS/GA/TA/AgNPs (3-Ag).

The FTIR spectra of the controls and target composites are presented in Figure 4a,b. The spectra show the general characteristics of the functional groups of CS, which is the most abundant component in all the samples. The broad band at ~3000–3600 cm^−1^ corresponds to the stretching of hydroxyl groups and N-H groups, while -CH stretching is observed at the lower wavenumber region, at ~2700–3000 cm^−1^, N-H bending appears in the region ~1300–1700 cm^−1^ and the symmetric stretching of C-O-H, C-O-C and C-N-H groups are found at approximately 900–1200 cm^−1^ [46,47]. Whereas the main features appear in the same areas with the introduction of additional components to CS, some changes in the exact wavenumbers of the bands are observed. For example, in the region from 1024 to 1075 cm^−1^, peaks associated with -CH-OH, -CH_2_-OH and other bonds (deriving from skeletal vibrations involving C-O stretching) were shifted to higher frequency values. Upon the addition of crosslinking agents, GA and/or TA, the first peak, which is most likely associated with C-O-C functionality, shifted from 1025 cm^−1^ for the neat CS samples to 1027, 1027 and 1032 cm^−1^ for the CS/GA/TA, CS/TA/AgNP and CS/GA/TA/AgNP composites, respectively. The neighboring band for the CS control sample was located at 1067 cm^−1^, while for the most complex composite it appeared to be blue shifted at 1074.2 cm^−1^. This is a direct indication that the crosslinking event has been successful and that complexation and bond formation at different levels had taken place in all the cases where TA, GA and AgNPs were present. Moreover, when TA is present (CS/GA/TA), one more broad peak attributed to C=O functional group is observed at 1700 cm^−1^; when AgNPs were added to the very same system (CS/GA/TA/AgNPs), the peak disappeared due to the formation of additional bonds and the creation of a tighter molecular structure in terms of the polymer matrix, which was also observed by TEM. We observed a slightly different pattern in another spectral region, 1420–1377 cm^−1^. According to the references, these are the typical absorption bands, corresponding to the coupling of C-N axial stretching and N-H angular deformation [48,49]. For the silver-containing samples (the CS/AgNPs, CS/TA/AgNPs and CS/GA/TA/AgNPs), we observed the appearance of the band at 826 cm^−1^, which most likely corresponds to the interaction of silver with oxygen-rich groups [34,50].

#### 2.2.2. Scanning Electron Microscopy

The morphology and homogeneity of the colloidal nanocomposites were investigated by means of scanning electron microscopy (SEM).

**Figure 5:**
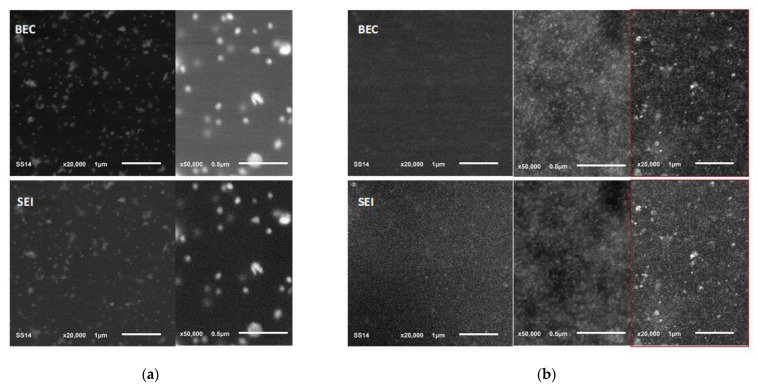
SEM lower (×20,000) and higher (×50,000) magnification images of the colloidal solutions deposited on the Si substrate: (**a**) CS/TA/AgNPs (2-Ag) and (**b**) CS/GA/TA/AgNPs (3-Ag) with the 6-months-old stability control sample highlighted in red. Backscattered electron (BEC) and secondary electron (SEI) detectors were used to acquire images.

The morphology and homogeneity of the colloidal nanocomposites were investigated by means of SEM. The surface images of the deposited colloidal solutions on Si wafer substrate indicate that all the composites have evenly distributed nanoparticles: 2-Ag (Figure 5a) has homogeneously distributed individual nanoparticles and a relatively large number of aggregates, which appear brighter within the matrix. In the case of 3-Ag, the presence of incorporated metal nanophases is less obvious at a lower magnification, but they are still visible (Figure 5b). Nevertheless, in Figure 5b, even at a low magnification (×20,000), a faint signal is seen coming from the metal NPs. The signal weakness is most likely due to the abundance of the polymer matrix and to the ultrafine size of the particles: the tight polymer network being formed around small sized AgNPs. Moreover, at a higher magnification it is possible to observe evenly distributed small AgNPs within the polymer matrix. It is also possible to observe the porous structure of the polymer itself at a magnification of ×50,000. The stability of the most complex colloidal solution was also investigated, revealing a stronger signal coming from the metal core of the 6-months-old composite, which is highlighted in red in Figure 5b. This might be attributed to the slow time-related decrease in the stability of the core-shell composite and the increase in the number of non-stabilized NPs as well as the further formation of a higher number of aggregates, which is in accordance with the stability test results made by UV-vis spectroscopy and TEM analysis.

#### 2.2.3. DLS and ζ-Potential Measurements

**Figure 6:**
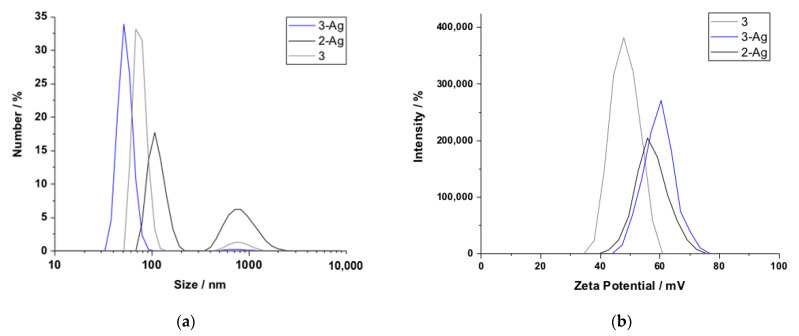
Results of (**a**) colloidal size distribution measurements by DLS and (**b**) ζ-Potential measurements for the colloidal solutions: CS/GA/TA/AgNPs (3-Ag) in blue, CS/TA/AgNPs (2-Ag) in dark grey, control CS/GA/TA (3) in light grey.

Several estimations on the size and dispersity of composites of different compositions were revealed using dynamic light scattering (DLS) data. The hydrodynamic diameter (HDD) represents the size of a solvated particle, most often with a complex nanocomposite structure: an entrapped metal core in the organic shell. The HDDs of the colloids found by DLS has higher values than those measured by TEM: DLS provides us both the size of the hydrated polymer blend shell and the incorporated nanosilver core, whereas it is possible to measure only the high-electron density metal core, with only a partial view of the shell and its possible thickness at the desiccated status, using TEM. Interestingly, even without presence of a metal core, the CS/GA/TA polymer blend is able to form particles with the average HDD of 83 nm, with these being lower in size than CS/TA/AgNPs (126 nm) and bigger in size than CS/GA/TA/AgNPs, with an average of 60 nm (Figure 6a). HDD and corresponding PDI values are reported in Supplementary Material Table S1 in accordance with the previous statements found by UV-vis and TEM: when GA is present, 3-Ag, the polymer blend with incorporated AgNPs, has a higher stability, with a PDI below 0.4, whereas 2-Ag and the pure polymer blend give values of 0.47 and 0.54, respectively.

To use colloids as a potential antipathogen agent, it is important to determine their stability at different pH values. As a rule of thumb, a zeta potential value that is higher or lower than + or −30 mV is considered to have enough repulsive force to maintain physical stability. Supplementary Material Table S1 presents the ZP values of fresh (acidic pH ~6) and treated colloidal samples (pH 7.2). The ZP results for the fresh 2-Ag and 3-Ag were found to be +57.4 mV and +60.3 mV, respectively (Figure 6b). The control sample, without the presence of a metal core, also exhibited a positive surface charge, with this being +48.6 mV. High absolute zeta potential values signal good colloidal stability, with the highest value corresponding to the sample containing GA. To understand the influence and real aggregation state of the colloidal solution, the diluted samples were filtered through a 220 nm pore size filter. The resulting surface charge of the CS/TA/AgNPs changes from +57.4 mV to +27.6 mV, signaling a drastic drop in stability. In case of the most complex composite, the drop in ZP was not as significant. Concerning UV-vis spectra of colloidal solutions before and after filtration, as presented in Supplementary Material Figure S1, the presence of the SPR peak for both 2-Ag and 3-Ag is observed. Interestingly, after filtration, it was possible to observe the AgNPs plasmon only in case of the GA-containing sample. This might be related to the fact that AgNPs are tightly immobilized in aggregates of the polymer matrix, whilst the other sample (3-Ag) has NPs in a supported state, both individually and agglomerated, but not aggregated. In other words, all the particles of 2-Ag were left on the filter; meanwhile, in the case of 3-Ag, some of them were deposited on the filter and some of them went through it. In order to study stability of the core-shell nanoparticles, the particles’ ZP was investigated at pH 7 (Supplementary Material Figure S2), which is the pH of culture media for microbiological experiments. The ZP dropped significantly: from 57.4 mV to 37 mV for 2-Ag and from 60.3 mV to 29.3 mV for 3-Ag. High absolute surface charge values indicate good stability even at a higher pH, at which the antibiofilm experiments were carried out.

### 2.3. Antibiofilm Tests of Core-Shell Nanocolloids

#### 2.3.1. Biofilm Development in the Presence of and without Treatment with Nanocolloids

**Figure 7:**
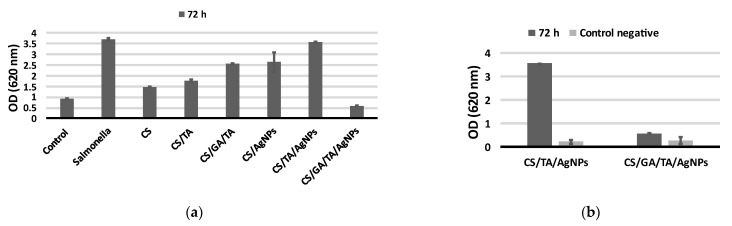
Graphical representation of the optical density (OD) of *Salmonella enterica* biofilms and control samples after 72 h incubation attached to the bottom of individual wells in 96-well microplate (**a**) at 620 nm derived from the ethanol elution of the crystal violet stain; (**b**) comparison of OD values of treated biofilm and control negative measurements of polymer blend without bacteria for the same incubation conditions. Error bars represent statistical analysis based on triplicate measurements.

An investigation of the antibiofilm properties of synthesized colloidal solutions was carried out against *Salmonella enterica* (SE) and *Listeria monocytogenes* (LM). The inhibition of biofilm formation depends directly on the compositions of the colloidal solutions and the abundance of the NPs. Graphs with OD values at different wavelengths after a crystal violet (CV) assay are shown in the Figure 7. There is a clear trend in terms of potential inhibition of SE bacteria and biofilm growth: the OD rises with the increase in composite complexity. It is only for the CS/GA/TA/AgNPs (3-Ag) that the OD value is in the same range as the control, corresponding to full biofilm growth inhibition. To be sure that the CV is not staining the nanocomposite, and the polysaccharide-based polymer shell in particular, control experiments for incubated colloids in the culture medium without bacteria were carried out (Figure 7b). It was demonstrated that the negative control samples of both the CS/TA/AgNPs (2-Ag) and CS/GA/TA/AgNPs have a negligible interaction with the CV, with the value being almost the same as for the blank control.

**Figure 8:**
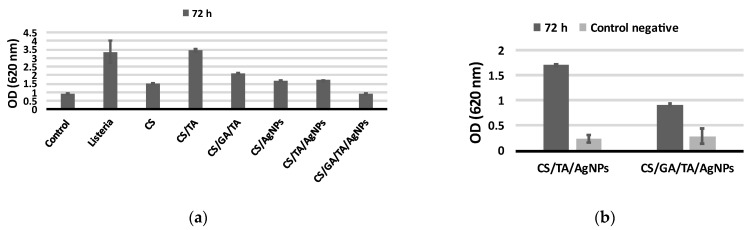
Graphical representation of the optical density (OD) of *Listeria monocytogenes* biofilms and control samples after 72 h incubation attached to the bottom of individual wells in a 96-well microplate (**a**) at 620 nm derived from the ethanol elution of the crystal violet stain; (**b**) comparison of OD values of treated biofilm and control negative measurements of polymer blend without bacteria for the same incubation conditions. Error bars represent statistical analysis based on triplicate measurements.

In case of Gram-positive LM, the results differ from those for SE (Figure 8). There is no clear trend in terms of potential inhibition, the OD values are higher than for the control in all the cases and in some cases these reach the OD value of the LM control sample. As in the previous case, with Gram-negative SE, we see an OD value for the CS/GA/TA/AgNPs (3-Ag) sample in the same range as the control negative without bacteria, indicating biofilm growth inhibition, shown in Figure 8. The differences in results might be attributed to the differences in the membrane structures of Gram-positive and Gram-negative bacteria [51]. In this case, a more detailed examination of the mechanism of interaction of the bacterial membrane with the composite components and composites of different compositions is needed.

#### 2.3.2. Direct Observation of Biological Samples with Confocal Laser Scanning Microscopy (CLSM)

**Figure 9:**
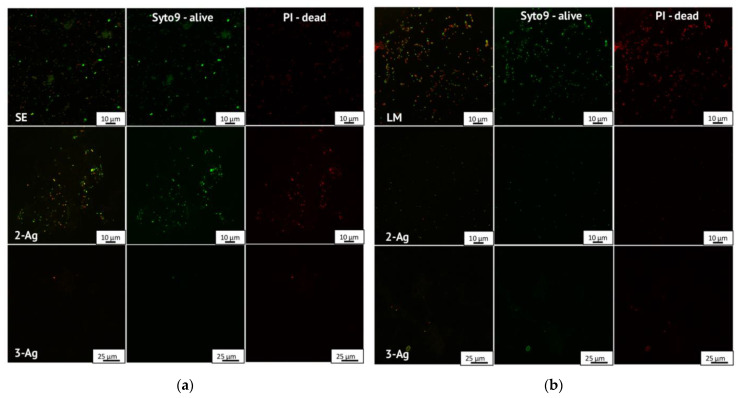
Visualization of biofilms tagged with fluorophores after 72 h incubation under CLSM: (**a**) *Salmonella enterica SE* control, treated with CS/TA/AgNPs (2-Ag) and treated with CS/GA/TA/AgNPs (3-Ag); (**b**) *Listeria monocytogenes LM* control, treated with 2-Ag and treated with 3-Ag. Represented in green—tagged with SYTO9—are alive bacteria, in red—tagged with Propidium Iodide (PI)—are dead bacteria, and merged images of green and red signals are also shown.

Using CLSM it was possible to visualize the presence of bacteria, which were tagged with fluorophores depending on their state: the bacteria appeared in green when alive and in red when dead, and an overlay of the two states together was also introduced for comparative reasons (Figure 9). In Figure 9, control SE (a) and LM (b) samples are shown, as are those treated with CS/TA/AgNPs (2-Ag) and CS/GA/TA/AgNPs (3-Ag). In case of SE, it is obvious that bacteria are present in the alive state in the cases of the control and those treated with 2-Ag but are absent in case of the most complex composition of nanocomposites, 3-Ag. It should be mentioned that when bacteria are treated with 2-Ag, they have time to start forming a biofilm/adhering to the surface, but they start dying during the incubation, which is when they appear as yellow and red on the merged image in Figure 9a 2-Ag. In case of LM (Figure 9b), both 2-Ag and 3-Ag appeared to prevent the formation of the biofilm, resulting in absence of detectable bacteria.

**Figure 10:**
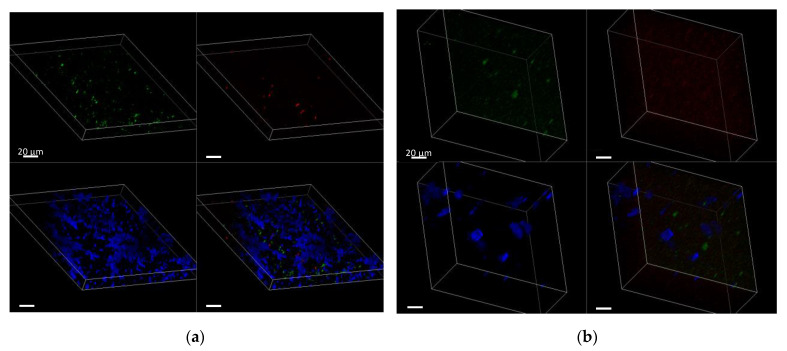
3D reconstruction of biofilm after incubation of: (**a**) control SE sample and (**b**) SE sample treated with CS/ TA/AgNPs (2-Ag). Represented in green—tagged with SYTO9—are alive bacteria, in red—tagged with propidium iodide (PI)—are dead bacteria and in blue—tagged with Alexa 647—is the extracellular matrix (ECM). The scalebar corresponds to 20 μm.

When it comes to biofilm formation, there is one important and unique feature which has to be investigated. In order to investigate presence of the extracellular matrix (ECM), a unique feature of the biofilm structure, another fluorescent dye (Alexa 647) was used. On the 3D images reconstructed in Figure 10, the ECM appears in blue with a relatively high abundance on the examined surface of the SE control sample (Figure 10a), while for the sample treated with 2-Ag, examining an even thicker area of interest, the ECM formation is lower and less obvious (Figure 10b). For the most complex composition with the highest crosslinking degree, the biofilm formation and presence of the ECM was not observed. This serves as further evidence of the effectiveness of the nanocomposites against the formation of the biofilm of SE, in accordance with the CV assay results.

#### 2.3.3. Observation of Morphological Changes after Incubation

In order to investigate possible biofilm formation and bacterial immobilization during the incubation within the polymer network of the nanocomposite, SEM was used.

**Figure 11:**
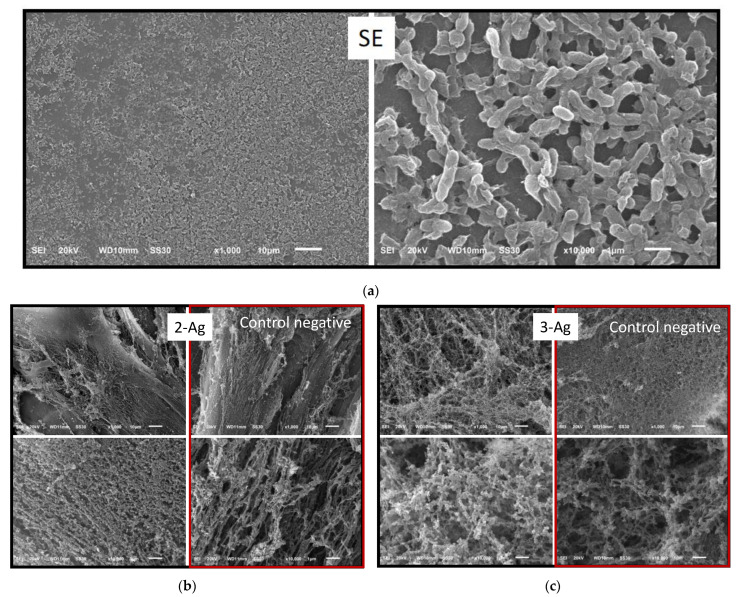
SEM of bacterial samples after incubation with and without treatment with colloidal solutions and control negative solutions (without bacteria) to observe differences in morphology: (**a**) control SE samples without treatment, (**b**) SE and control negative (highlighted in red) treated with CS/TA/AgNPs (2-Ag) and (**c**) SE and control negative (highlighted in red) treated with CS/GA/TA/AgNPs (3-Ag).

By observing differences in terms of morphologies between the control bacterial sample, the treated samples and their control negatives (culture medium without bacteria), it was possible to correlate the data with the CLSM data. In Figure 11a we can see that the control sample for SE has the ability to form a strong enough biofilm and bacteria are tightly immobilized within the network, with them being able to withstand the preparation procedure. In the case of 2-Ag, in Figure 11b, there is a significant morphological difference between the sample of interest and the control negative (highlighted in red). No bacteria were observed within the polymer network, which might be related to the thorough preparation procedure for SEM analysis. On the other hand, 3-Ag showed no significant changes in terms of morphology at higher magnification after the incubation with (Figure 11c) and without bacteria. This way, it was demonstrated that when the most complex nanocomposition, 3-Ag, is used, biofilm formation is inhibited. All the results are in accordance with the data obtained by CV assay, the OD values and by CLSM.

## 3. Materials and Methods

### 3.1. Chemical Synthesis of Core-Shell Nanocolloids

Chitosan with a medium molecular weight and a 75–85% deacetylation degree and a purified glutaraldehyde solution (grade I) of 25% in H_2_O were used. All the other chemicals were of analytical grade and purchased from Sigma-Aldrich (Merck Group), Darmstadt, Germany.

### 3.2. UV-vis Spectroscopy

UV-vis spectra of the as-prepared diluted composites were recorded using a Shimadzu UV-1601 spectrophotometer in the 300–800 nm range in quartz cuvettes, Hellma Analytics (Müllheim, Germany). The presented results were obtained by averaging three single measurements.

### 3.3. Transmission Electron Microscopy (TEM)

Transmission electron microscopy (FEI Tecnai 12 TEM, Eindhoven, Netherlands) equipped with a LaB6 filament operating at 120 kV), was used to measure the AgNPs’ size distribution and to study the morphology of the composites. For this analysis, the TEM samples were prepared by placing several microliters of diluted solutions of CS/TA/AgNPs and CS/GA/TA/AgNPs on polymer coated copper grid (Formwar coated, 300 mesh, Agar Scientific). Samples were thoroughly dried in air and subsequently transferred to the microscope. Size distribution analysis was performed with Fiji-Image J software (version 2.0.0-rc-69/1.52i), and the number of analyzed particles was more than 120.

### 3.4. Fourier Transform Infrared Spectroscopy (FTIR)

The Fourier transform infrared (FTIR) spectra of the solutions were recorded on a Perkin Elmer Spectrum Two FTIR spectrophotometer (PerkinElmer, Inc., Waltham, MA, USA) with a DTGS detector in transmittance mode (scan number 32, resolution 2 cm^−1^, range 4000–400 cm^−1^) and processed using the Spectrum software (version 10.5.4). Several microliters of the control and colloidal solutions were placed on the crystal and air dried.

### 3.5. Dynamic Light Scattering (DLS) and Z-Potential Measurements

The hydrodynamic radii R_h_, dispersity of core-shell nanocomposites and zeta potential measurements were measured using a Nano ZS from Zetasizer, Malvern Instruments (Malvern Instruments, Malvern, UK), operating with a 4mW He-Ne laser (633 nm) at a constant 25°. The hydrodynamic radius was measured by the instrument using dynamic light scattering (DLS) performed in backscattering at a fixed detection angle of 173°. The size distribution by number was recovered using the software implemented by the manufacturer by taking the inverse Laplace transform of the ACF and the subsequent application of the Stokes–Einstein equation assuming a medium viscosity 0.8872 mPa s; medium refractive index 1.330. The zeta potential measurements were performed using laser Doppler electrophoresis through phase analysis light scattering (LDE-PALS) using a forward scattering (17°) setup in disposable capillary cells. Some of the colloidal solutions were measured and then filtered through a 0.2 μm membrane filter for an additional measurement. Several samples were brought from their original pH (~6) to the pH of culture medium (7.2) by the addition of sodium carbonate dropwise to understand the stability during the antimicrobial experiments.

### 3.6. Scanning Electron Microscopy (SEM)

The morphology and homogeneity in terms of metal nanophase dispersion within the organic matrix were investigated using a JEOL-6610LV Scanning Electron Microscope, Tokyo, Japan. Fresh and in some cases aged colloidal solutions were deposited on clean Si wafer substrate, air dried and investigated under an electron beam with an accelerated voltage of 20 kV (Tungsten filament). Images were acquired using two detectors: backscattered (BEC) and secondary electrons (SEI) were used.

In case of *Salmonella enterica*, the morphology of the biofilm structure after incubation with and without treatment was investigated by means of SEM. The sample preparation was as follows: Si wafer was deposited on the bottom of the microplate before the introduction of bacterial suspension and/or treatment and carefully extracted after incubation and a washing procedure. The fixation was carried out in 2.5% GA solution. Then, the serial dehydration (70, 80, 90, 100 %) was carried out in EtOH. Samples were air dried and subjected to gold sputtering.

### 3.7. Development of Monomicrobial Biofilms

For this study, the common foodborne pathogens *Salmonella enterica* CECT 4594 and *Listeria monocytogenes* CECT 935 were selected. Those bacteria were included in antibiofilm protocol in order to evaluate activity against both Gram-positive and Gram-negative species. Bacterial stocks were cultured on tryptic soy agar (TSA), and the plates were incubated at 37 °C for 24 h. After that, isolated single colonies of the microorganisms were routinely grown overnight in Tryptic Soy Broth (TSB) medium at 37 °C.

To study biofilm formation and antibiofilm protocols, the microtiter plate assay was used. Bacterial suspension was prepared in TSB, with a final microbial concentration of 10^8^ CFU/mL, by adjusting 0.5 OD at 550 nm, followed by serial dilution to the final concentration of 10^6^ CFU/mL. In this method, 100 µL of bacteria were incubated for 72 h in a microtiter polystyrene 96-well plate at 37 °C in static conditions with and without the introduction of 20 µL of the colloidal treatments of different compositions (Supplementary Material Figure S3a). Following incubation, each plate was rinsed with Milli-Q water to eliminate planktonic bacteria and subjected to the direct visualization and crystal violet staining assay. Control negative samples were introduced to eliminate the effect of side reactions: TSB medium without bacteria was combined with colloidal solutions for incubation and further treatment at the same conditions as the bacterial suspensions incubated with colloidal solutions. 

### 3.8. Confocal Microscopy (CLSM)

For the direct visualization of bacterial species, confocal laser scanning microscopy (CLSM) was used. A working solution of propidium iodide (PI) and SYTO 9 dye mixtures (FilmTracer™ LIVEDEAD Biofilm Viability kit, Invitrogen, Waltham, MA, USA) and Alexa Fluor 647 (ThermoFisher Scientific, Waltham, MA, USA) were prepared according to the instructions of the providers. The excitation settings were 500/530, 655/750 and 650/671 nm, respectively. The biofilms were washed with sterile phosphate-buffered saline (PBS) and transferred to a microscopy glass slide. The 24 mm × 50 mm coverslips were placed on work area and were stained with 6 µL dye mixture and incubated for 5 min. Oil immersion was used on the coverslips, and the micrographs were recorded with CLSM (Leica SP8) at the University of Oviedo, Oviedo, Spain.

### 3.9. Crystal Violet (CV) Assay and Optical Density (OD) Measurements

For staining with crystal violet (CV) dye, a solution 150 mL of 2% crystal violet in 95% ethanol was introduced to the rinsed microplates for 1 h. These were then washed thoroughly three times in fresh Milli-Q water and left to air dry (Supplementary Material Figure S3b). Then, 200 mL of 70% ethanol solution was introduced to each microplate to recover the dye from the biofilm. The optical densities were measured at a wavelength of 620 nm using the microplate reader Multiscan EX, Thermo Scientific with Ascent 2.6 software.

## 4. Conclusions

In this work, extremely stable core-shell nanocomposites with spherical silver nanophases as a core were developed using silver nitrate as a precursor and natural compounds as additives/coadjuvants, with a fast and scalable chemical synthesis. Both chitosan and tannic acid, in the presence of glutaraldehyde, were used in a crosslinked polymer blend to reduce and stabilize AgNPs with a size of 17 ± 4 nm, creating a protective organic shell of 2.7 nm and providing prolonged stability for the nanocolloids. The influence of the presence of the different components of polymer blend on the nanoparticles’ size distribution, stability and antibiofilm properties was studied by using microscopic and spectroscopic methods. The most complex composition, with a highly crosslinked polymer blend, the CS/GA/TA/AgNPs, showed significant antibiofilm activity against *Salmonella enterica* and *Listeria monocytogenes* biofilms: when introduced to the bacterial suspension, the colloidal solution prevented the formation of biofilms. Therefore, the synthesized colloidal nanocomposites could potentially be used for the further creation of novel non-conventional antimicrobial and antibiofilm materials. A comparison of the performance of the colloidal nanocomposites with conventional antibiotics and their potential synergy could be made to prevent AMR development. Taking into account the film forming properties of the polymer blend, this liquid colloidal material could be used as a starting point for the creating of solid forms, for example biodegradable films.

## Figures and Tables

**Table 1:** Labels for the samples, using different reducing and stabilizing agents.

Composition of the Nanocomposite: Stabilizing and Reducing Agent(s) and Presence of AgNPs	Sample Label
CS	1
CS/TA	2
CS/GA/TA	3
CS/AgNPs	1-Ag
CS/TA/AgNPs	2-Ag
CS/GA/TA/AgNPs	3-Ag

**Table 2:** Size of the AgNPs and thin organic shells around the core for different polymer blend compositions.

Sample Name	Size of AgNPs, nm	SD	Organic Shell Thickness, nm	SD	SPR Max, nm
**CS/TA/AgNPs**	18	5	5.5	0.6	445
**CS/GA/TA/AgNPs**	17	4	2.7	0.1	420

## Data Availability

The data presented in this study are available on request from the first and the corresponding authors.

## Supplementary Materials

The following supporting information can be downloaded at: https://www.mdpi.com/article/10.3390/antibiotics11101396/s1, Table S1: Summary of the AgNPs SPR position maxima, HDD found by DLS with corresponding PDI and Zeta Potentials with corresponding SD for the fresh and treated by pH adjustment and filtration samples. Figure S1: (a) DLS measurements and (b) UV-visible spectra before and after filtration of the fresh colloidal solutions. Upper photo corresponds to the CS/GA/TA/AgNPs sample, lower to CS/TA/AgNPs. Figure S2. Zeta Potential measurements for fresh and treated (adjustment of pH to 7). Figure S3. 96-well microplates visual appearance (a) after incubation and (b) after CV assay.
