# Supplementary material for: Highly Stable Core-Shell Nanocolloids: Synergy between Nano-Silver and Natural Polymers to Prevent Biofilm Formation
Source: Antibiotics (Basel). 2022 Oct 12;11(10):1396. doi: 10.3390/antibiotics11101396 (PMC9598106; doi:10.3390/antibiotics11101396)
Supplement: Supplementary file 1 [file antibiotics-11-01396-s001.zip › antibiotics-1960413-supplementary.pdf]

antibiotics

MDPI

Article

Highly Stable Core-Shell Nanocolloids: Synergy between Nano-Silver and Natural Polymers to Prevent Biofilm Formation

Ekaterina A. Kukushkina [1,2], Helena Mateos [1,2], Nazan Altun [3], Maria Chiara Sportelli [1], Pelayo Gonzalez [3], Rosaria Anna Picca [1,2], Nicola Cioffi [1,2*]

Supplementary Information

Table S1. Summary of the AgNPs SPR position maxima, HDD found by DLS with corresponding PDI, and Zeta Potentials with corresponding SD for the fresh and treated by pH adjustment and filtration samples.

Sample name	SPR max, nm	HDD, nm	PDI	ZP, mV	SD
**CS/GA/TA - control**	no SPR	82.6	0.542	48.6	0.635
**CS/GA/TA/AgNPs**	420	60.4	0.342	60.3	0.656
**CS/TA/AgNPs**	≈455	124.5	0.473	57.4	0.208
**CS/GA/TA – control filtr**	no SPR	51.3	0.531		
**CS/GA/TA/AgNPs filtr**	423	58.2	0.472	50.7	0.153
**CS/TA/AgNPs filtr**	no SPR	30.9	0.392	27.6	*4.53*
**CS/GA/TA/AgNPs (pH 7)**	420	83.4	0.287	29.3	0.52
**CS/TA/AgNPs (pH 7)**	≈470	70.5	0.283	37.3	0.265

Figure S1. a) DLS measurements and b) UV-visible spectra before and after filtration of the fresh colloidal solutions. Upper photo corresponds to the CS/GA/TA/AgNPs sample, lower to CS/TA/AgNPs.

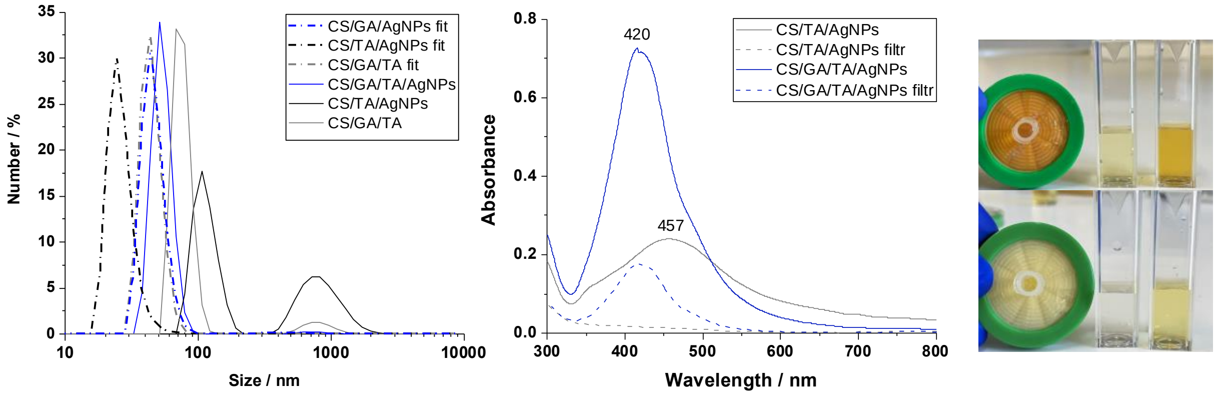

(a) **(b)**

Figure S2. Zeta Potential measurements for fresh and treated (adjustment of pH to 7).

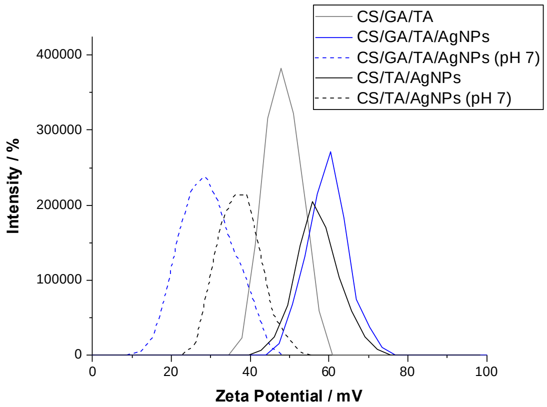

Figure S3. 96-well microplates visual appearance a) after incubation and b) after CV assay.

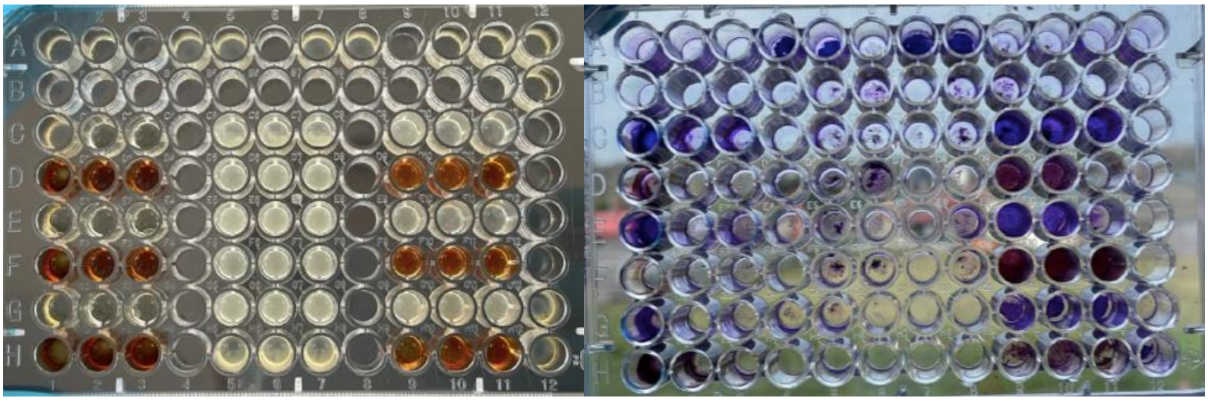

(a) (b)
